# Immunocyte-derived extracellular vesicles in osteoimmunology: mechanisms, disease contexts, and translational prospects

**DOI:** 10.3389/fimmu.2025.1709048

**Published:** 2025-11-24

**Authors:** Lixuan Yang, Ning Li, Hao Xu, Jinman Chen, Qianqian Liang

**Affiliations:** 1 Longhua Hospital, Shanghai University of Traditional Chinese Medicine, Shanghai, China; 2 Spine Institute, Shanghai University of Traditional Chinese Medicine, Shanghai, China; 3 School of Traditional Chinese Medicine, Shanghai University of Traditional Chinese Medicine, Shanghai, China; 4 Key Laboratory of Ministry of Education of Theory and Therapy of Muscles and Bones, Shanghai University of Traditional Chinese Medicine, Shanghai, China; 5 State Key Laboratory of Discovery and Utilization of Functional Components in Traditional Chinese Medicine, Shanghai, China

**Keywords:** extracellular vesicles, osteoimmunology, bone remodeling, macrophages, rheumatoid arthritis, osteoporosis, periodontitis, biomaterial-integrated EVs

## Abstract

Bone remodeling disorders such as osteoporosis, rheumatoid arthritis, and periodontitis highlight the clinical significance of osteoimmune communication. Osteoimmunology has emerged as a key interdisciplinary field elucidating the dynamic interplay between the immune and skeletal systems, with extracellular vesicles (EVs) recognized as nanosized mediators that transport proteins, lipids, and RNAs to regulate bone remodeling. Immunocyte-derived EVs modulate osteoblast and osteoclast activity through macrophage polarization, Treg-associated CD73/adenosine signaling, Th17/Treg balance, and B cell–bone interactions, exerting dual effects by promoting bone formation under physiological conditions while amplifying inflammation and bone resorption in osteoporosis, rheumatoid arthritis, and periodontitis. Bidirectional communication between bone marrow stromal cell–derived EVs and immune cells further highlights the complexity of EV-mediated regulation in bone microenvironments. Moreover, engineering approaches such as cargo loading, surface modification, and biomaterial integration are rapidly advancing the therapeutic application of EVs in bone diseases. Despite these advances, challenges remain in EV standardization, scalable production, and clinical translation, underscoring that immunocyte-derived EVs represent both pathogenic mediators and promising therapeutic agents, with future studies required to resolve mechanistic complexity and optimize their clinical utility. Engineered EVs enable targeted modulation of CD73–adenosine, NF-κB, HIF-1α, and PI3K/AKT axes, offering bone-targeting delivery and immune-instructive biomaterials as converging strategies. These insights highlight immunocyte-derived EVs as both biomarkers and therapeutic candidates in bone disorders, and underscore the need for standardized approaches to advance their clinical utility in osteoimmunology.

## Introduction

Osteoimmunology is a rapidly evolving interdisciplinary field that investigates the complex bidirectional interactions between the skeletal and immune systems. It elucidates the fundamental molecular mechanisms governing both bone homeostasis and pathological conditions (1). This intricate crosstalk is particularly prominent in bone remodeling, where immune cells and their secreted cytokines directly modulate osteoblast and osteoclast activity. Extracellular vesicles (EVs) have recently emerged as essential nanoscale mediators in this intercellular communication network, facilitating the transfer of bioactive molecules between immune and bone cells (2, 3).

These membrane-bound nanoparticles, typically 40–160 nm in diameter, transport diverse molecular cargo—including proteins, lipids, mRNAs, and microRNAs (miRNAs)—, that critically regulate bone formation, resorption, angiogenesis, and immune responses within the bone microenvironment (2, 4).

The pathological relevance of EV-mediated communication is evident in multiple diseases. In periodontitis, Porphyromonas gingivalis–derived EVs suppress acetylcholine secretion by inhibiting Cyp4f40, which accelerates osteoclastogenesis and bone loss (5). Similarly, immunocyte-derived EVs facilitate bone metastasis by establishing pre-metastatic niches through integrin-mediated homing and microenvironmental modulation (6). Collectively, these studies indicate that EVs are central regulators maintaining skeletal balance under physiological conditions, but when dysregulated, they contribute to osteoporosis, rheumatoid arthritis, and periodontitis (7–9).

The bone microenvironment is increasingly recognized as an EV-rich niche, where vesicle-mediated immune–bone crosstalk contributes to both skeletal development and pathological bone loss. Here, we first summarize the biology and cargo features of immunocyte-derived EVs, then outline their bidirectional crosstalk with bone marrow stromal cells (BMSCs), examine disease-specific roles in periodontitis, osteoporosis, and rheumatoid arthritis, and finally discuss engineering and translational applications.

## Biology and cargo of immunocyte-EVs

EVs are lipid-bilayer particles secreted by virtually all cell types. According to the Minimal Information for Studies of Extracellular Vesicles 2023 (MISEV2023) guidelines, “EVs” is the preferred generic term encompassing particles previously referred to as exosomes or microvesicles, which differ mainly by size and biogenesis. Accordingly, EVs are broadly categorized as BMSC small EVs (sEVs; 40–160 nm, typically of endosomal origin), microvesicles (100–1000 nm, derived from plasma-membrane budding), and apoptotic bodies (1–5 µm, released during apoptosis) (2, 10). Their molecular cargo—comprising proteins, lipids, and nucleic acids—reflects the cell of origin, activation state, and microenvironmental cues, thereby mediating intercellular communication under both physiological and pathological conditions (11–13). This heterogeneity accounts for the broad functional spectrum of EVs in skeletal homeostasis and pathological bone remodeling.

Dendritic-cell-derived EVs (DC-EVs) exemplify the immunomodulatory breadth of this system. Mature DC-EVs act as potent mucosal adjuvants that enhance both systemic and local immune responses (14). Beyond immunostimulation, alterations in circulating EV profiles offer clinically relevant biomarkers: in rheumatoid arthritis, CD14^+^ HLA-DR^+^ EVs reflect antigen-presenting-cell activation (15), and plasma RANKL^+^ EVs correlate with bone metastases and skeletal-related events (16).

Macrophage-derived EVs (Mφ-EVs) are central mediators of bone remodeling. Their actions depend on polarization: M1-EVs amplify inflammatory cascades and osteoclastogenesis, whereas M2-EVs resolve inflammation and enhance angiogenic–osteogenic coupling (13, 17). Mechanical and chemical cues—including lithium-doped calcium-silicate scaffolds, cyclic stretch, and shear stress—reshape EV cargo such as miR-145-5p, mitochondrial components, and miR-423-5p, thereby promoting osteogenic differentiation of bone-marrow stromal cells (13, 18, 19). M2-EVs also suppress osteoclastogenesis through the PKM2/HIF-1α axis (20). Erythropoietin-stimulated macrophages secrete miR-5107-5p-rich EVs that inhibit EGFR and restore osteogenesis under inflammatory stress (21). Electrical preconditioning enriches oxidative-phosphorylation proteins within macrophage EVs, further potentiating their regenerative activity (22). Collectively, M2-EVs exert pro-regenerative and anti-inflammatory effects, whereas M1- or pathogen-modified EVs intensify osteoclastic activation and bone resorption (13, 17–22). Conversely, adipose-tissue macrophages under estrogen deficiency release pro-inflammatory small EVs carrying miR-30e-5p that reinforce M1 polarization (23), whereas Porphyromonas gingivalis gingipains suppress miR-146a-5p in BMSC-EVs, activating TRAF6 and driving osteoclastogenesis (24).

Lymphocyte-derived EVs constitute another immunoregulatory axis in bone remodeling. Retinoic-acid-induced Treg EVs enriched in CD73 hydrolyze AMP to adenosine, suppressing IL-17A and RANKL expression and thereby alleviating alveolar bone loss in periodontitis (9). In rheumatoid arthritis, pro-inflammatory CD80^+^ macrophages sustain immune imbalance; EVs engineered to deliver methotrexate reprogram them toward anti-inflammatory phenotypes, enhance Treg differentiation, and suppress Th1 responses (25). B-cell-derived EVs add an additional layer of immune control. BMSC-EVs induce B10 regulatory-cell differentiation without excessive IL-10 production, indicating fine-tuned B-cell tolerance (26).

Professional antigen-presenting-cell EVs further contribute to the oste-immune network, and circulating EV signatures serve as accessible biomarkers. Collectively, immunocyte-derived EVs convey cell-of-origin-specific cargo with context-dependent effects, acting as bidirectional modulators of bone formation and resorption. Representative cargo and target pathways are summarized in **Table 1**.

**Table 1:** Summary of key studies on immunocyte-derived EVs and bone remodeling.

Immune cell source	Representative EV cargo/markers	Target cells/pathways	Functional effects	References
Macrophage (M1)	miR-30e-5p, pro-inflammatory proteins	Osteoclast precursors, inflammatory milieu	Promote inflammation, drive osteoclastogenesis	(17, 20, 23, 24)
Macrophage (M2)	miR-145-5p, mitochondria-rich vesicles, oxidative phosphorylation proteins	BMSCs (osteogenesis), angiogenic pathways	Enhance osteogenesis, angiogenesis, and anti-inflammatory responses	(13, 18, 19, 21, 22)
Treg cells	CD73^+^ EVs (adenosine-producing)	Osteoclasts, effector CD4^+^ T cells	Suppress inflammation, reduce RANKL/IL-17A, protect bone	(9, 25)
B cells	BMSC-EVs promoting B10 phenotype	Breg subsets (CD1d^+^CD5^+^, IL-10^+^CD45^+^)	Induce regulatory phenotype without IL-10 upregulation	(26)
Dendritic cells	Mature DC-EVs, antigen-presenting markers (CD14, HLA-DR)	Mucosal/systemic immunity, bone immune microenvironment	Act as mucosal adjuvants, modulate systemic and local immune tone	(14–16)

This table lists representative studies describing the roles of immunocyte-derived extracellular vesicles (EVs) in bone remodeling, highlighting their cellular sources, cargo molecules, signaling pathways, and functional outcomes in osteoclasts and osteoblasts.

## BMSC-EVs: reciprocal crosstalk with immune cells

EV-mediated communication between immune cells and BMSCs constitutes a bidirectional regulatory axis within the bone microenvironment. Inbound M2 macrophage–derived EVs enhance BMSC osteogenic differentiation, whereas inflammatory EVs suppress lineage commitment and promote catabolic signaling. Outbound BMSC-EVs (CD73^+^/PD-L1^+^) attenuate excessive macrophage and T-cell activation; however, chronic TNF-α or IFN-γ stimulation reduces these immunosuppressive subsets (12). In diabetes, BMSC-EVs exhibit diminished CD73 expression, weakening their capacity to promote M2 polarization and bone formation. Conversely, CD73^+^ BMSC-EVs activate A2b-adenosine receptor and cAMP signaling to restore osteogenesis (27).

Reciprocal regulation by immune cells also shapes the BMSC-EV landscape. VPS33B-dependent EV secretion mediates autocrine signaling in BMSCs, and disruption of this pathway accelerates cellular senescence, bone loss, and remodeling imbalance (28). Importantly, transplantation of EVs from young BMSCs restores osteogenic potential and mitigates age-related skeletal decline. Therapeutically, engineered BMSC-EVs overexpressing CTLA4Ig or IL-1 receptor antagonist exhibit potent immunomodulatory effects in collagen-induced arthritis, increasing TGF-β1 expression and suppressing IL-6 and RANTES levels, thereby alleviating inflammation and promoting bone repair (29).

Collectively, BMSC-EVs act as editors of immune tone within bone niches, integrating inbound immune cues and releasing outbound immunoregulatory cargo. Dysregulation of this bidirectional communication contributes to impaired regeneration and disease progression, whereas targeted engineering of BMSC-EVs provides a promising avenue for osteo-immune modulation and skeletal regeneration (12, 27–29).

## Pathogenic roles of immunocyte-EVs in bone diseases

With the mechanistic toolkit in place, these principles can be mapped onto specific disease contexts. Immunocyte-derived EVs act as double-edged mediators in bone diseases, functioning either as pathogenic drivers or as therapeutic agents depending on their cellular origin and cargo composition (3, 30).

### Periodontitis

Oral infection and local dysbiosis reshape EV traffic at barrier tissues, tipping the balance toward osteoclastogenesis and inflammatory bone loss. Infection-associated EVs play a central role in periodontal bone loss. Porphyromonas gingivalis infection of oral keratinocytes induces a tenfold increase in EV release, enriching vesicles with TNF-α and IL-1β that exacerbate local inflammation and tissue damage (31). Gingipains further disrupt skeletal balance by downregulating miR-146a-5p in BMSC-derived EVs, thereby activating TRAF6 and promoting osteoclastogenesis (24). In estrogen-deficient conditions, adipose macrophages secrete pro-inflammatory sEVs (e.g., miR-30e-5p) that amplify M1-like polarization and worsen disease severity (23).

Therapeutic EVs demonstrate potential for regeneration. M2 macrophage-derived EVs reprogram immature neutrophils into Anxa1^+^ subsets, promoting inflamed bone repair (17). Likewise, erythropoietin-stimulated macrophage EVs deliver miR-5107-5p to BMSCs, suppress EGFR, and restore osteogenesis in inflammatory settings (21). Engineered CD80-targeting EVs carrying methotrexate selectively suppress pro-inflammatory macrophages, enhance Treg differentiation, and reduce osteoclastogenesis (25). Collectively, these findings underscore the dual role of EVs in periodontitis progression and therapy. Thus, in periodontitis, EVs can either accelerate alveolar bone destruction or serve as regenerative mediators depending on their cellular origin.

### Osteoporosis

Systemic aging, endocrine factors, and microbiota-derived signals converge on EV programs that weaken bone mass and repair capacity. In osteoporosis, immunocyte- and microbiota-derived EVs contribute to bone fragility through multiple mechanisms. In glucocorticoid-induced disease, miR-370-3p regulates the TLR4/SLC7A11/GPX4 axis to enhance osteogenesis while inhibiting ferroptosis, thereby attenuating bone loss (32). Senescent cell accumulation accelerates skeletal decline, whereas engineered bifunctional EVs that promote both senolysis and efferocytosis show anti-aging therapeutic benefits (33). Gut microbiota-derived EVs also mediate the gut–bone axis, linking microbial dysbiosis to osteoporosis risk (34, 35).

Nutritional and plant-derived EVs provide additional protective effects. Milk-derived vesicles activate BMP2/MAPK pathways and enhance osteogenesis (36, 37). Plant EVs from Epimedium and sea buckthorn stimulate bone regeneration via PI3K/AKT and miRNA-mediated signaling, respectively (38, 39). These studies underscore that EVs from senescent cells, microbiota, and dietary sources collectively influence osteoporosis pathogenesis and therapy. Together, these studies highlight how diverse EV sources influence osteoporosis pathogenesis and may be harnessed for intervention.

These observations emphasize that diverse EV sources—from senescent cells to dietary vesicles—can both compromise and restore skeletal integrity.

### Rheumatoid arthritis

Autoimmune inflammation reprograms joint and circulating EVs, coupling synovitis to cartilage and bone damage. Distinct EV signatures have been identified in rheumatoid arthritis (RA). Synovial fibroblast-derived sEVs carrying miRNA-15-29148 induce chondrocyte apoptosis via CIAPIN1 targeting, contributing to joint degeneration (40). Circulating EVs from RA patients are enriched in antigen-presenting cell markers such as CD14 and HLA-DR, reflecting immune activation within inflamed joints (15).

Therapeutically, probiotic-derived EVs favor M2 polarization and suppress pro-inflammatory cytokines, ameliorating disease activity (8, 41). Engineered EVs co-loaded with methotrexate and CD80 antibodies further suppress inflammatory macrophages and enhance Treg responses, illustrating the translational potential of immune-targeted vesicles (25).

### Other orthopedic diseases: ONFH and OA/TMJ-OA

Beyond osteoporosis, rheumatoid arthritis, and periodontitis, accumulating evidence has linked bone-marrow-mesenchymal-stem-cell-derived extracellular vesicles (BMSC-EVs) to additional orthopedic disorders. In osteonecrosis of the femoral head (ONFH), BMSC-EVs enriched with miR-148a-3p suppress SMURF1, enhance osteogenic differentiation, and mitigate disease progression (42). In osteoarthritis, including temporomandibular-joint OA (TMJ-OA), sEVs facilitate cartilage reconstruction through the autotaxin–YAP signaling axis, reducing inflammation and restoring joint architecture (43). These findings extend the osteo-immune relevance of BMSC-EVs beyond inflammatory bone loss to degenerative skeletal disorders, highlighting SMURF1 and YAP as potential therapeutic targets.

**Figure 1:**
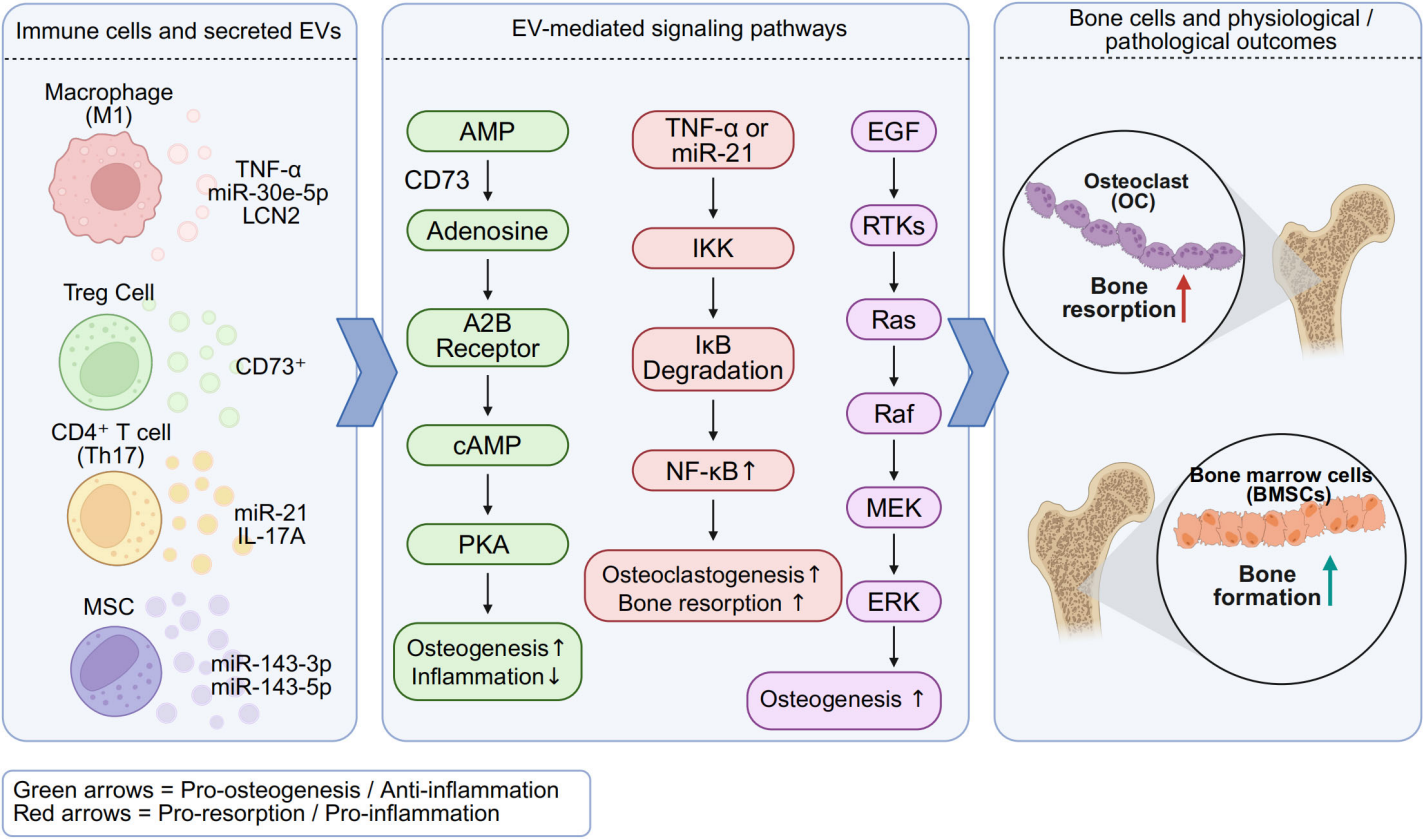
Osteoimmune signaling by immunocyte-derived EVs in bone remodeling. Schematic representation of cargo-specific pathways summarized in **Table 2**. Macrophage- and T cell-derived EVs carry distinct miRNAs and proteins that regulate osteoclastogenesis and osteogenesis via CD73–adenosine, NF-κB, and HIF-1α signaling. M1-EVs enriched in TNF-α and miR-21 promote inflammation and osteoclast activity, whereas M2-EVs and Treg-derived EVs carrying CD73 facilitate immunosuppression and osteoblast differentiation. These EV-mediated pathways shape the osteoimmune microenvironment and contribute to skeletal homeostasis or pathological bone loss. Created with BioRender.com.

Taken together, across bone diseases—including periodontitis, osteoporosis, rheumatoid arthritis, and degenerative entities such as ONFH and OA—immunocyte-derived EVs act as double-edged mediators: pathogenic EVs amplify inflammatory osteoclastogenesis, whereas regenerative or engineered EVs restore osteo-immune balance. Representative pathogenic and therapeutic roles are summarized in **Table 2**, and **Figure 1** schematizes the cargo-specific signaling circuits through which EVs shape bone remodeling outcomes.

**Table 2:** Representative pathogenic and therapeutic roles of immunocyte-derived EVs across major bone diseases.

Disease	Pathogenic EVs	Therapeutic EVs	Mechanisms	References
Periodontitis	P. gingivalis EVs (gingipains), adipose macrophage sEVs (miR-30e-5p)	M2-EVs, EPO-stimulated macrophage EVs, engineered methotrexate-loaded EVs	Promote vs. suppress osteoclastogenesis; regulate EGFR and TRAF6 pathways	(23–25, 31)
Osteoporosis	Senescent-cell EVs, microbiota-derived EVs	Milk-derived EVs, plant-derived EVs (Epimedium, sea buckthorn), engineered bifunctional EVs	Regulate ferroptosis (TLR4/SLC7A11/GPX4); modulate osteogenesis and bone mass	(32–39)
Rheumatoid arthritis	Synovial fibroblast EVs (miRNA-15-29148), antigen-presenting cell EVs (CD14, HLA-DR)	Probiotic EVs, engineered methotrexate-loaded EVs	Induce chondrocyte apoptosis, sustain inflammation vs. promote immunomodulation	(8, 15, 25, 40, 41)
ONFH/OA (TMJ-OA)	ONFH: BMSC-EVs deficient in miR-148a-3p → up-regulate SMURF1 → impaired osteogenesis; OA (TMJ-OA): Inflammatory EVs disrupt cartilage homeostasis	BMSC-EVs enriched with miR-148a-3p or small EVs activating autotaxin–YAP signaling	Promote osteogenic differentiation and cartilage reconstruction; suppress SMURF1 and inflammatory signaling	(42, 43)

**Table 2** summarizes representative pathogenic and therapeutic roles of immunocyte-derived extracellular vesicles (EVs) across major bone diseases, including degenerative disorders (ONFH and OA). Examples are illustrative rather than exhaustive.

### Engineering and translational applications

These mechanistic and disease insights motivate engineering strategies to control EV cargo, targeting, and release. Advanced engineering approaches enhance the therapeutic utility of immunocyte-derived EVs by improving targeting specificity, sustaining release, and boosting bioactivity (44–48). These strategies mainly involve cargo loading, surface modification, and integration with biomaterials.

Hypoxia preconditioning of magnesium-activated dental pulp stem cells enriches EVs with miR-451a. These vesicles promote angiogenesis through the AKT/eNOS/NO axis (49, 50).Surface modification. Decorating EV membranes improves tissue specificity. FNDC5/irisin-enriched EVs conjugated with bone-targeting aptamers efficiently accumulated in skeletal tissues and alleviated osteoporosis (50). Antibody-modified EVs, such as CD80-coated vesicles, selectively targeted inflammatory macrophages and enhanced therapeutic outcomes in RA and periodontitis (25). Similarly, bacterial EVs engineered to express DC-STAMP directly delivered inhibitory peptides into osteoclast precursors, suppressing osteoclastogenesis in osteoporosis models (47). Surface activation strategies, such as choline phosphate modification, further improve EV binding efficiency to biomaterials (48). Surface modification enables precise tissue targeting *in vivo*. Such strategies enable precise tissue targeting and improve therapeutic specificity.

Biomaterial integration. Incorporation of EVs into scaffolds enhances retention and controlled release. GelMA hydrogels provide a supportive 3D matrix for EV delivery, improving bone regeneration efficiency (38, 51). Thermosensitive hydrogels constructed from polyhedral oligomeric silsesquioxane, PEG, and PPG effectively carried aspirin-treated macrophage EVs, promoting osteochondral repair (52). Electrospun membranes incorporating lipocalin-2–enriched EVs accelerated healing of large bone defects (53). Likewise, 3D-printed porous titanium scaffolds combined with stem cell EVs and anchored by zwitterionic coatings improved osteogenic repair (48). Biomaterial integration improves EV stability and prolongs functional activity. These systems stabilize EVs and provide spatiotemporal control of release *in vivo*.

Hybrid platforms. Integrative systems combine cargo engineering, surface targeting, and biomaterial encapsulation for synergistic benefits. For example, an Exo@Tβ4/HAMA hydrogel system, generated by grafting thymosin β4 onto modified hyaluronic acid and encapsulating BMSC-EVs (Exo@Tβ4 refers to thymosin β4-modified HAMA hydrogels loaded with EVs, promoted BMSC recruitment, neurogenesis, angiogenesis, and osteogenesis (54). Similarly, dual biomimetic scaffolds that integrated 3D-printed Ti-6Al-4V frameworks with EVs-loaded PEGDA/GelMA microspheres enabled sustained release and enhanced regenerative outcomes (55).

Collectively, engineering of EV cargo, surfaces, and biomaterial integration provides a clear trajectory toward clinical translation by improving specificity, stability, and spatiotemporal control of osteo-immune modulation.

### Emerging technologies enabling EV-based targeted osteoimmune modulation

Recent advances in multi-omics and bioengineering are transforming EVs research from descriptive biology into translationally actionable platforms.

#### Single-EV and spatial multi-omics

Bulk analyses mask EV heterogeneity. Single-EV proteomics and transcriptomics now permit high-resolution profiling of immunocyte-derived EVs, identifying distinct signatures in macrophage, T-cell, and B-cell subsets. Spatial transcriptomics further delineates EV deposition within bone marrow and inflamed synovium, directly mapping EV-mediated osteoimmune interactions *in situ* (56).

#### Artificial intelligence and machine learning

High-dimensional EV cargo datasets (miRNAs, proteins, lipids) challenge interpretation. AI-based classifiers stratify rheumatoid arthritis (RA) patients by plasma EV signatures and predict flare risk, suggesting applicability to osteoporosis or periodontitis (57, 58). Such computational tools accelerate biomarker discovery and support clinical translation of EV diagnostics.

#### Immune checkpoint–associated EVs

Beyond canonical CD73–adenosine signaling, checkpoint molecules are increasingly detected in EV cargo. Programmed death-ligand 1 (PD-L1)–positive EVs suppress T-cell activation and modulate local bone remodeling by shifting the Th17/Treg balance, linking immunotherapy-relevant pathways to skeletal disease (59, 60). These data position EVs as vehicles and targets for checkpoint modulation in osteoimmunology.

#### Exercise-mimetic and bone-targeted EVs

Skeletal muscle–derived irisin (FNDC5)–enriched EVs reproduce exercise-induced osteogenesis. Conjugation with bone-targeting aptamers enhances skeletal accumulation and attenuates bone loss in preclinical osteoporosis models (61, 62). Such exercise-mimetic EV platforms represent precision therapeutics for patients unable to exercise.

#### Standardization and regulatory alignment

Translation requires adherence to the Minimal Information for Studies of Extracellular Vesicles (MISEV2023) guidelines, emphasizing multimethod characterization, functional validation, and *in vivo* uptake (63, 64). In parallel, scalable good manufacturing practice (GMP)–compliant EV production and transparent reporting standards and harmonization with MISEV2023 are essential for reproducibility and regulatory approval (65). To facilitate clinical translation, **Table 3** summarizes key quality dimensions of EV research, aligning minimal experimental standards (MISEV2023) with preferred criteria for translational applications.

**Table 3:** Translational readiness checklist for EV-based osteoimmune therapeutics (aligned with MISEV2023).

Dimension	Minimal standard (MISEV2023) (63, 64)	Preferred for translation (65)	Examples in bone EV studies
Isolation & characterization	≥2 complementary methods; particle size and marker validation	Multi-platform validation; negative controls; contamination markers	RA plasma EV proteomics (58); MSC-EV nanoparticle tracking
*In vivo* assessment	Basic biodistribution and safety	Longitudinal kinetics, organ/bone tropism, immune readouts	Bone-targeting aptamer–EVs in osteoporosis (61)
Production & quality control	Laboratory-scale isolation	GMP-compatible scalable production, sterility and batch consistency	Engineered MTX-loaded EVs for RA (25, 57)
Regulatory & ethics	Source reporting, informed consent	Full traceability, regulatory pathway planning	Ongoing clinical EV trials in musculoskeletal repair (63)

The checklist highlights minimal experimental standards (as recommended by MISEV2023) and preferred criteria to enhance reproducibility and facilitate clinical application. Examples are drawn from recent bone EV studies, including macrophage- and Treg-derived EVs in periodontitis, bone-targeted aptamer-conjugated vesicles in osteoporosis, and engineered methotrexate-loaded EVs in rheumatoid arthritis.

## Conclusion and perspectives

Research on immunocyte-derived EVs has revealed their dual roles in bone remodeling, functioning as both pathogenic mediators and therapeutic agents. Despite rapid progress, several barriers still hinder clinical translation.

### Current limitations

Standardization of EV isolation and characterization remains a major challenge, with no universally accepted protocols and frequent heterogeneity across preparations (2, 66, 67). This undermines reproducibility and complicates cross-study comparisons. Scaling up production for clinical-grade applications is also technically demanding, as large-scale biomanufacturing must guarantee batch-to-batch consistency and stringent quality control (10, 68). These barriers highlight the urgent need for harmonized workflows across laboratories.

Although research on extracellular vesicles has expanded rapidly, several methodological and translational challenges remain unresolved. Heterogeneity in isolation procedures, quantification methods, and characterization markers continues to hinder reproducibility and cross-study comparison. Differences in parental cell sources, disease models, and culture conditions further complicate data interpretation. In most preclinical settings, evidence is derived from small-animal models with limited follow-up, and the long-term biodistribution, pharmacokinetics, and safety profiles of engineered EVs remain insufficiently defined. Moreover, the absence of unified potency assays and large-scale manufacturing standards restricts clinical translation. Future studies adhering to MISEV2023 recommendations, incorporating rigorous quality control, and integrating multicenter validation will be essential to establish reliable and clinically applicable EV-based osteoimmune therapies.

### Emerging opportunities

Advances in single-EV analysis, imaging, and multi-omics now provide unprecedented resolution of vesicle heterogeneity and disease-specific signatures (16). Circulating immunocyte-derived EVs show promise as accessible biomarkers for diagnosis and prognosis in osteoporosis, rheumatoid arthritis (RA), and periodontitis (3, 28). Concurrently, engineering approaches—including cargo loading, surface modification, and biomaterial integration—are enhancing the specificity and efficacy of EV-based therapeutics (45, 47).

### Future directions

Emerging frontiers such as single-EV omics and artificial-intelligence–driven biomarker discovery are expected to further refine mechanistic insight and diagnostic precision in osteoimmunology. Translation will require: (1) identification of key cargo molecules (miRNAs, proteins, lipids) that distinguish beneficial from pathogenic EVs; (2) implementation of standardized preparation, storage, and functional validation guidelines to ensure reproducibility (63, 69); and (3) combinatorial strategies integrating EV therapy with biomaterials, pharmacological agents, or cell-based interventions for synergistic efficacy, while addressing safety and regulatory concerns (70–73).

Taken together, immunocyte-derived EVs represent pivotal regulators of osteoimmunology, capable of driving both tissue repair and pathology. Advances in engineering and biomaterial integration provide strong momentum toward clinical application. Emerging platforms—including single-EV omics, artificial intelligence–driven biomarker discovery, and checkpoint- or exercise-mimetic vesicles—further expand the therapeutic landscape. With sustained collaboration among immunologists, bioengineers, and clinicians, these innovations delineate a realistic roadmap from mechanistic discovery to clinical translation.
